# Supplementary material for: The impact of phage and phage resistance on microbial community dynamics
Source: bioRxiv. 2023 Sep 26:2023.09.26.559468. Preprint. [Version 1] doi: 10.1101/2023.09.26.559468 (PMC10557685; doi:10.1101/2023.09.26.559468)
Supplement: Supplement 1 [file NIHPP2023.09.26.559468v1-supplement-1.pdf]

bioRxiv preprint doi: https://doi.org/10.1101/2023.09.26.559468; this version posted September 26, 2023. The copyright holder for this preprint (which was not certified by peer review) is the author/funder, who has granted bioRxiv a license to display the preprint in perpetuity. It is made available under aCC-BY 4.0 International license.

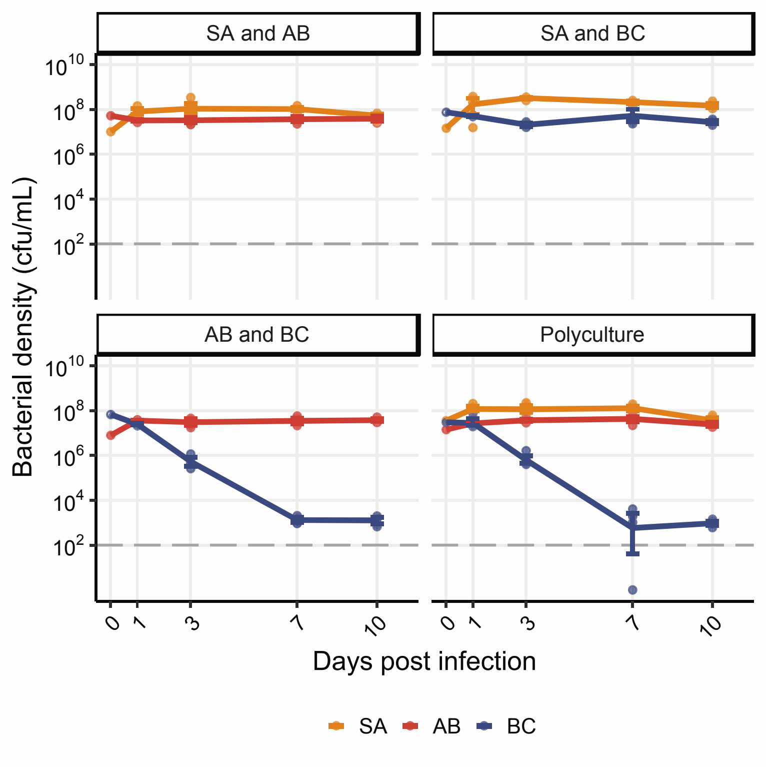

Supplemental Fig 1. Line plot of bacterial densities in the absence of *P. aeruginosa* and its phage. Showing the bacterial densities in cfu/mL over time for SA (*S. aureus*), AB (*A. baumannii*), and BC (*B. cenocepacia*) in various co-culture combinations in the absence of *P. aeruginosa* and its phage. Dashed horizontal line at 10^2 cfu/mL marks the threshold of reliable detection where the qPCR results indicate the bacteria has gone or is close to extinction from a population. Data are mean ± 95% CI.

bioRxiv preprint doi: https://doi.org/10.1101/2023.09.26.559468; this version posted September 26, 2023. The copyright holder for this preprint (which was not certified by peer review) is the author/funder, who has granted bioRxiv a license to display the preprint in perpetuity. It is made available under aCC-BY 4.0 International license.

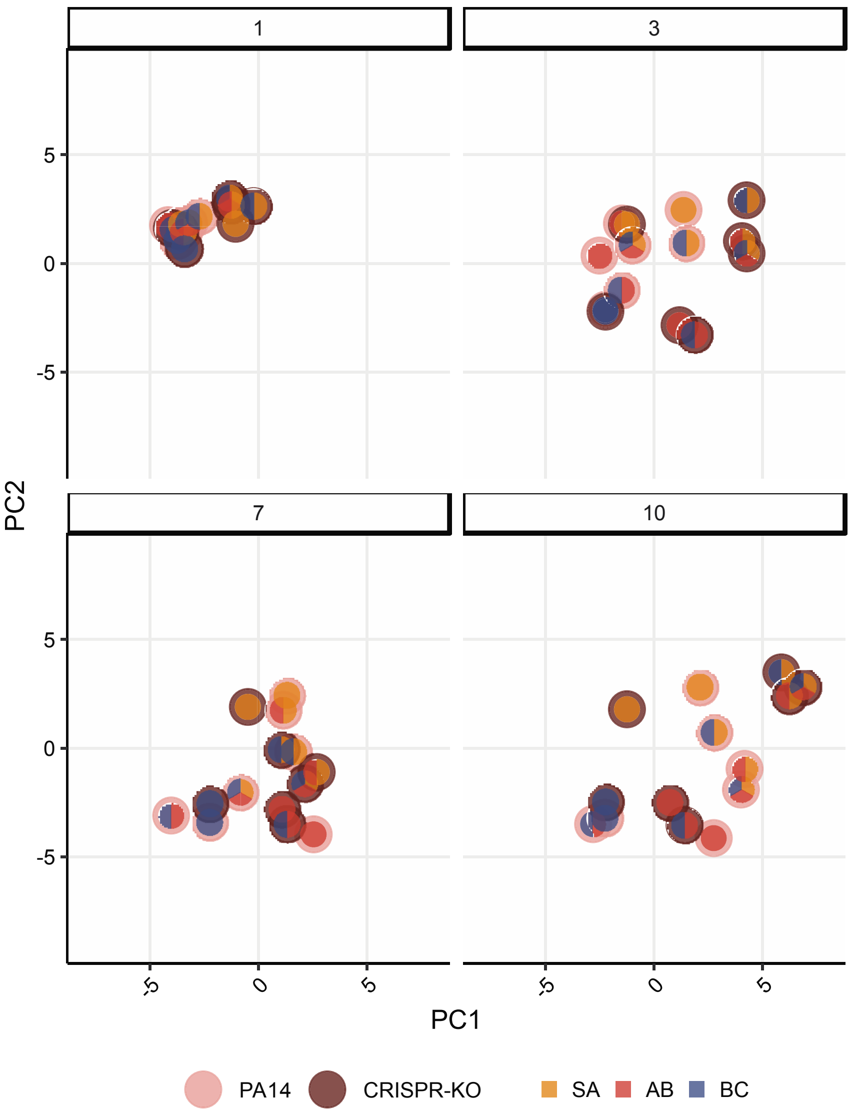

Supplemental Fig 2. Ordination plot in the absence of phage. PCA ordination of relative bacterial abundance in the absence of phage DMS3vir, with grid layouts separated into days post phage infection. Outer circle colour indicates which PA14 clone is present in the population, while inner circle indicates community composition (SA = *S. aureus*, AB = *A. baumannii*, BC = *B. cenocepacia*).

bioRxiv preprint doi: https://doi.org/10.1101/2023.09.26.559468; this version posted September 26, 2023. The copyright holder for this preprint (which was not certified by peer review) is the author/funder, who has granted bioRxiv a license to display the preprint in perpetuity. It is made available under aCC-BY 4.0 International license.

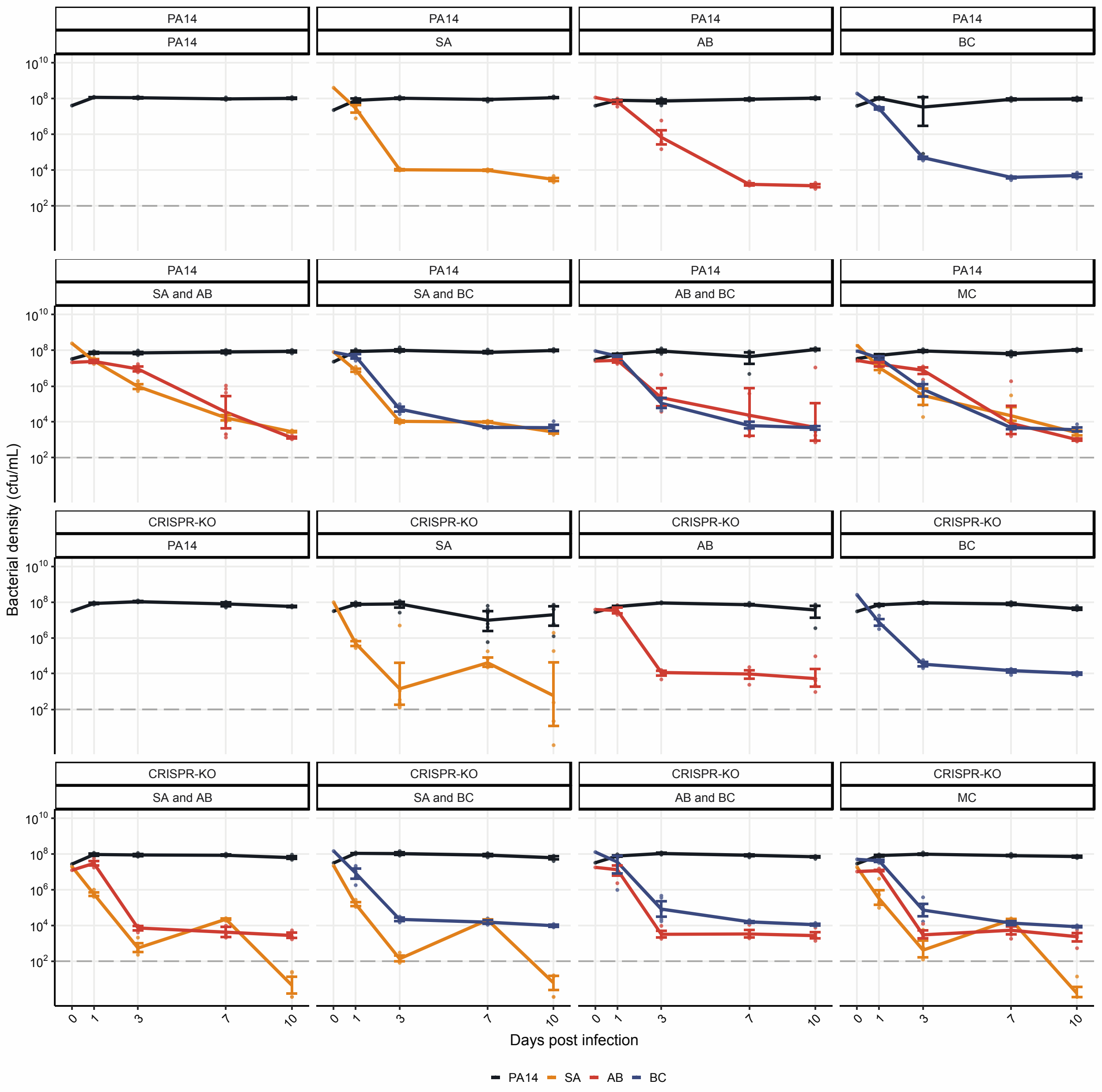

Supplemental Fig 3. Line plots of bacterial densities in the absence of phage. Showing the bacterial densities in cfu/mL over time for the PA14 WT and CRISPR-KO *P. aeruginosa* strains, and **b** the other microbial community species (SA = *S. aureus*, AB = *A. baumannii*, BC = *B. cenocepacia,* MC = microbial community) in the absence of phage DMS3vir. Dashed horizontal line at 10^2 cfu/mL marks the threshold of reliable detection

bioRxiv preprint doi: https://doi.org/10.1101/2023.09.26.559468; this version posted September 26, 2023. The copyright holder for this preprint (which was not certified by peer review) is the author/funder, who has granted bioRxiv a license to display the preprint in perpetuity. It is made available under aCC-BY 4.0 International license.

where the qPCR results indicate the bacteria has gone or is close to extinction from a population. Data are mean ± 95% CI.

bioRxiv preprint doi: https://doi.org/10.1101/2023.09.26.559468; this version posted September 26, 2023. The copyright holder for this preprint (which was not certified by peer review) is the author/funder, who has granted bioRxiv a license to display the preprint in perpetuity. It is made available under aCC-BY 4.0 International license.

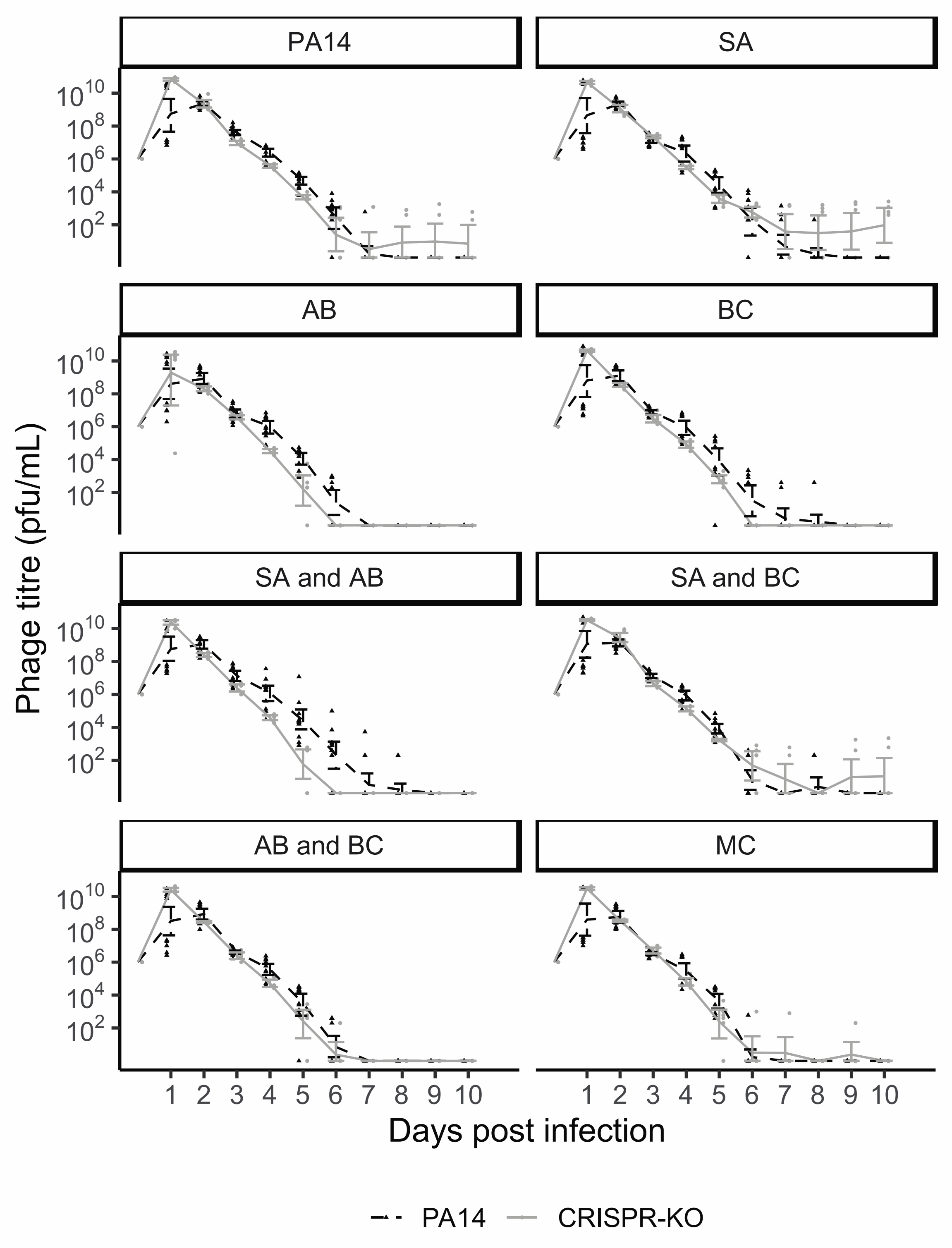

Supplemental Fig 4. Phage titres over time for each experimental treatment. Phage titres for phage DMS3vir over time across all experimental treatments (SA = *S. aureus*,

bioRxiv preprint doi: https://doi.org/10.1101/2023.09.26.559468; this version posted September 26, 2023. The copyright holder for this preprint (which was not certified by peer review) is the author/funder, who has granted bioRxiv a license to display the preprint in perpetuity. It is made available under aCC-BY 4.0 International license.

AB = *A. baumannii*, BC = *B. cenocepacia,* MC = microbial community), infecting either the PA14 WT or the CRISPR-KO strain as indicated by line type. Each data point represents a replicate, with lines following the mean and the error bars denoting 95% CI. Asterisks indicate a significant overall difference in phage density between the PA14 WT (n = 12 per timepoint) or CRISPR-KO clone (n = 6 per timepoint) (effect of *P. aeruginosa* clone; linear models: * $p < 0.05$).

bioRxiv preprint doi: https://doi.org/10.1101/2023.09.26.559468; this version posted September 26, 2023. The copyright holder for this preprint (which was not certified by peer review) is the author/funder, who has granted bioRxiv a license to display the preprint in perpetuity. It is made available under aCC-BY 4.0 International license.

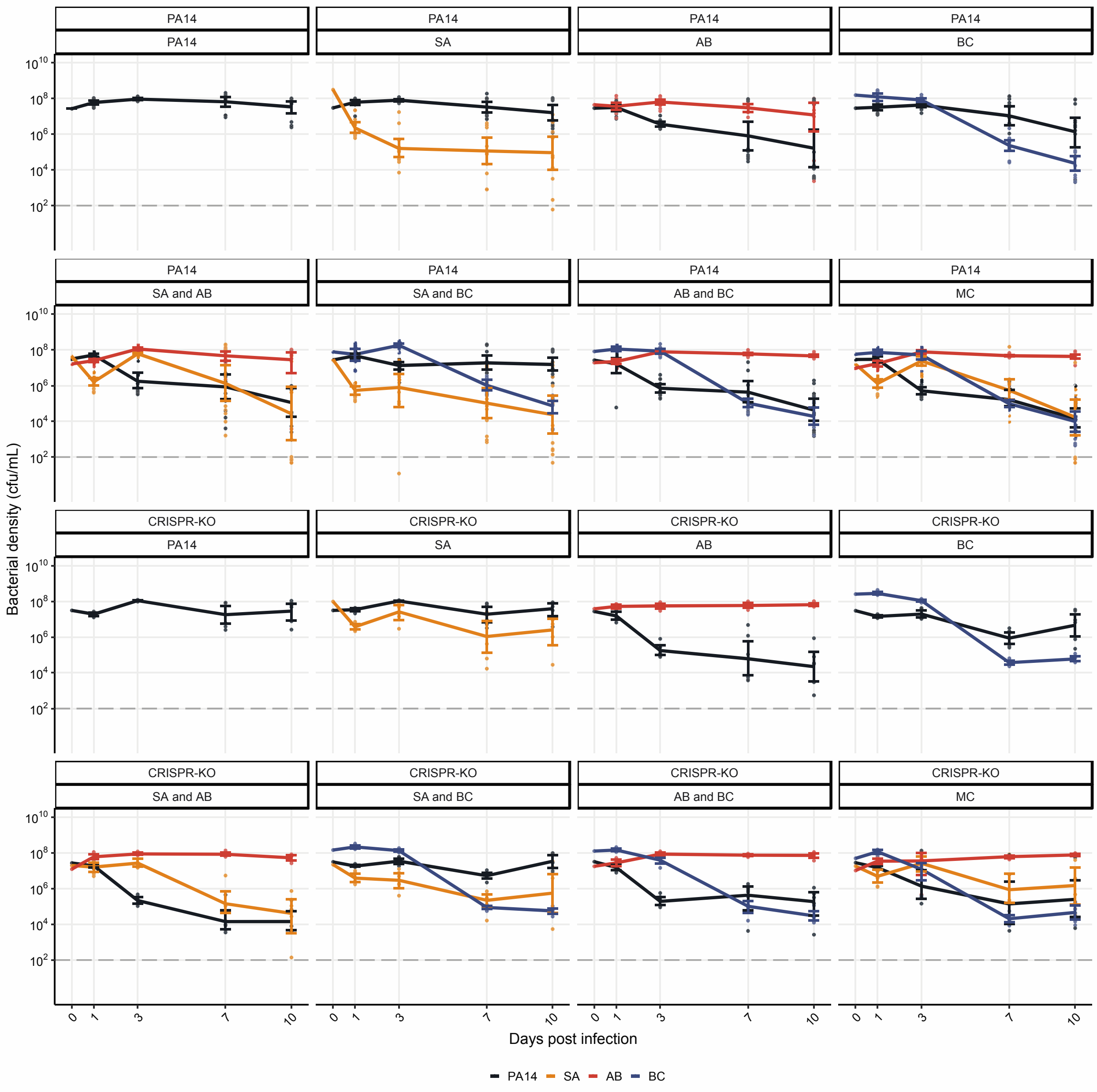

Supplemental Fig 5. Line plots of bacterial densities in the presence of phage. Showing the bacterial densities in cfu/mL over time for the PA14 WT and CRISPR-KO *P. aeruginosa* strains, and **b** the other microbial community species (SA = *S. aureus*, AB = *A. baumannii*, BC = *B. cenocepacia*, MC = Microbial community) in the presence of phage DMS3vir. Dashed horizontal line at 10^2 cfu/mL marks the threshold of reliable detection

bioRxiv preprint doi: https://doi.org/10.1101/2023.09.26.559468; this version posted September 26, 2023. The copyright holder for this preprint (which was not certified by peer review) is the author/funder, who has granted bioRxiv a license to display the preprint in perpetuity. It is made available under aCC-BY 4.0 International license.

where the qPCR results indicate the bacteria has gone or is close to extinction from a population. Data are mean ± 95% CI.

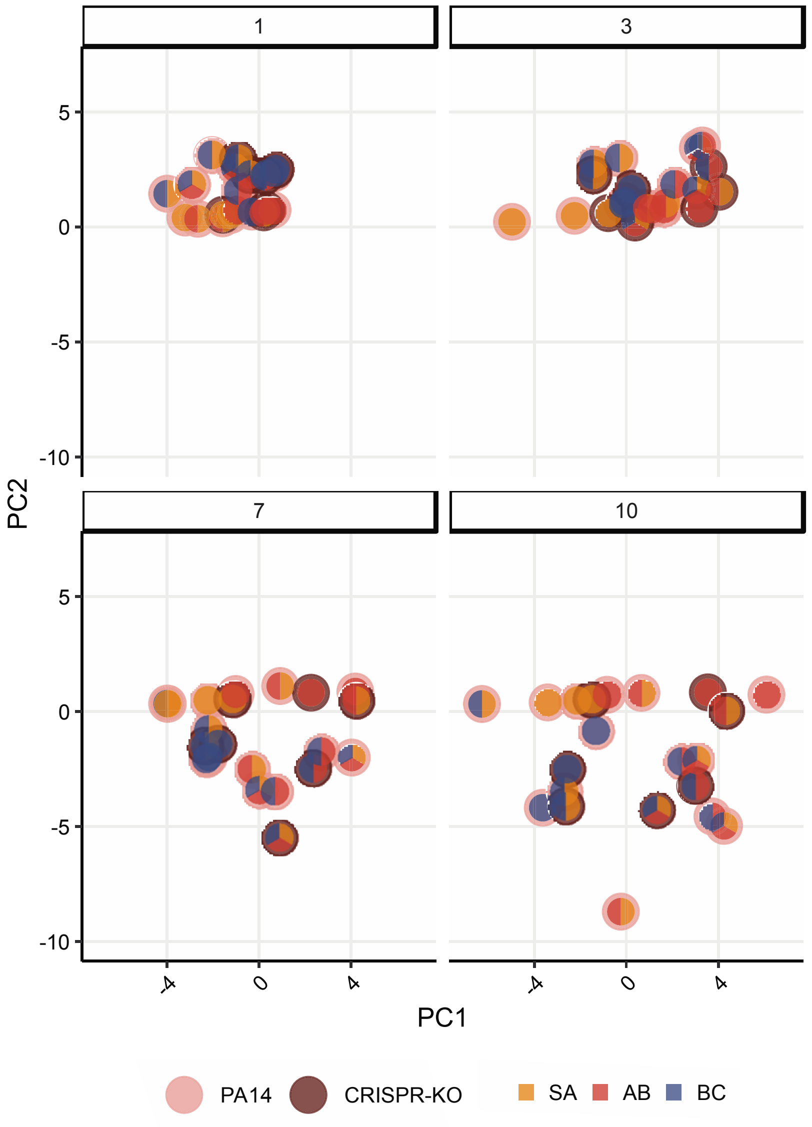

Supplemental Fig 6. Ordination plots in the presence of phage. PCA ordination of relative bacterial abundance in the presence of phage DMS3vir, with grid layouts separated into days post phage infection. Outer circle colour indicates which PA14 clone

bioRxiv preprint doi: https://doi.org/10.1101/2023.09.26.559468; this version posted September 26, 2023. The copyright holder for this preprint (which was not certified by peer review) is the author/funder, who has granted bioRxiv a license to display the preprint in perpetuity. It is made available under aCC-BY 4.0 International license.

is present in the population, while inner circle indicates community composition (SA = *S. aureus*, AB = *A. baumannii*, BC = *B. cenocepacia*).

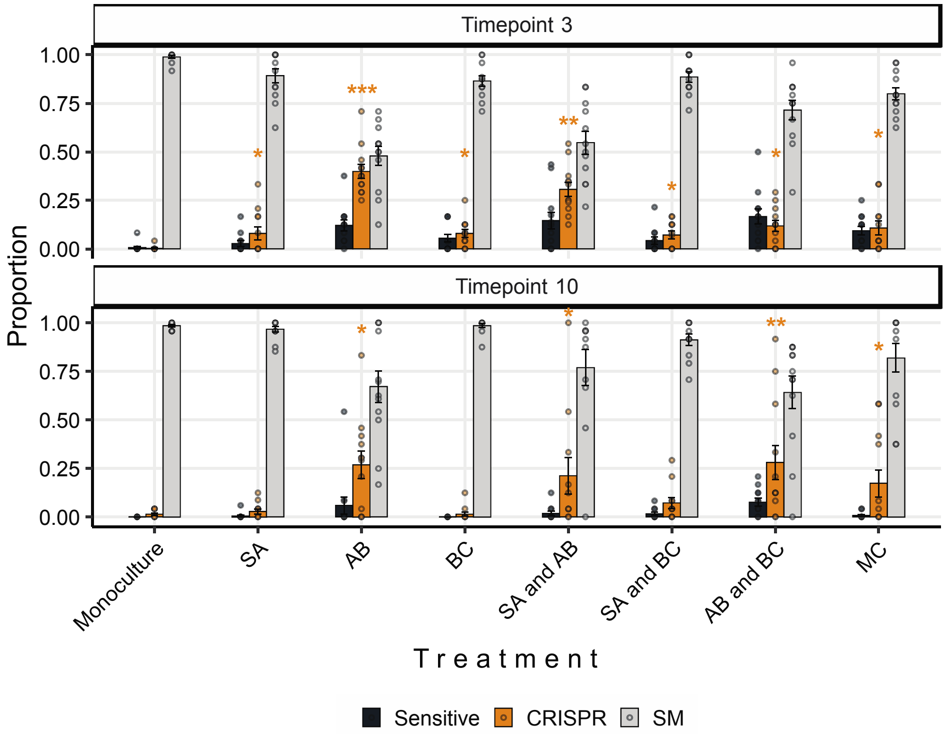

Supplemental Fig 7. Interspecific competition affects the proportion of evolved CRISPR-based phage resistance. Proportion of *P. aeruginosa* PA14 WT at timepoints 3 and 10 that evolved phage-resistance either through surface modification (SM) or CRISPR immunity, or which remained sensitive to phage DMS3vir when grown in monoculture or different polycultures (SA = *S. aureus*, AB = *A. baumannii*, BC = *B. cenocepacia*). Data are mean ± SE. Asterisks indicate a significant difference in proportion of CRISPR immunity evolved when compared to the PA14 monoculture within each timepoint (n = 12 per treatment) (generalised linear model, quasibinomial: * $p < 0.05$, ** $p < 0.01$, *** $p < 0.001$).

bioRxiv preprint doi: https://doi.org/10.1101/2023.09.26.559468; this version posted September 26, 2023. The copyright holder for this preprint (which was not certified by peer review) is the author/funder, who has granted bioRxiv a license to display the preprint in perpetuity. It is made available under aCC-BY 4.0 International license.

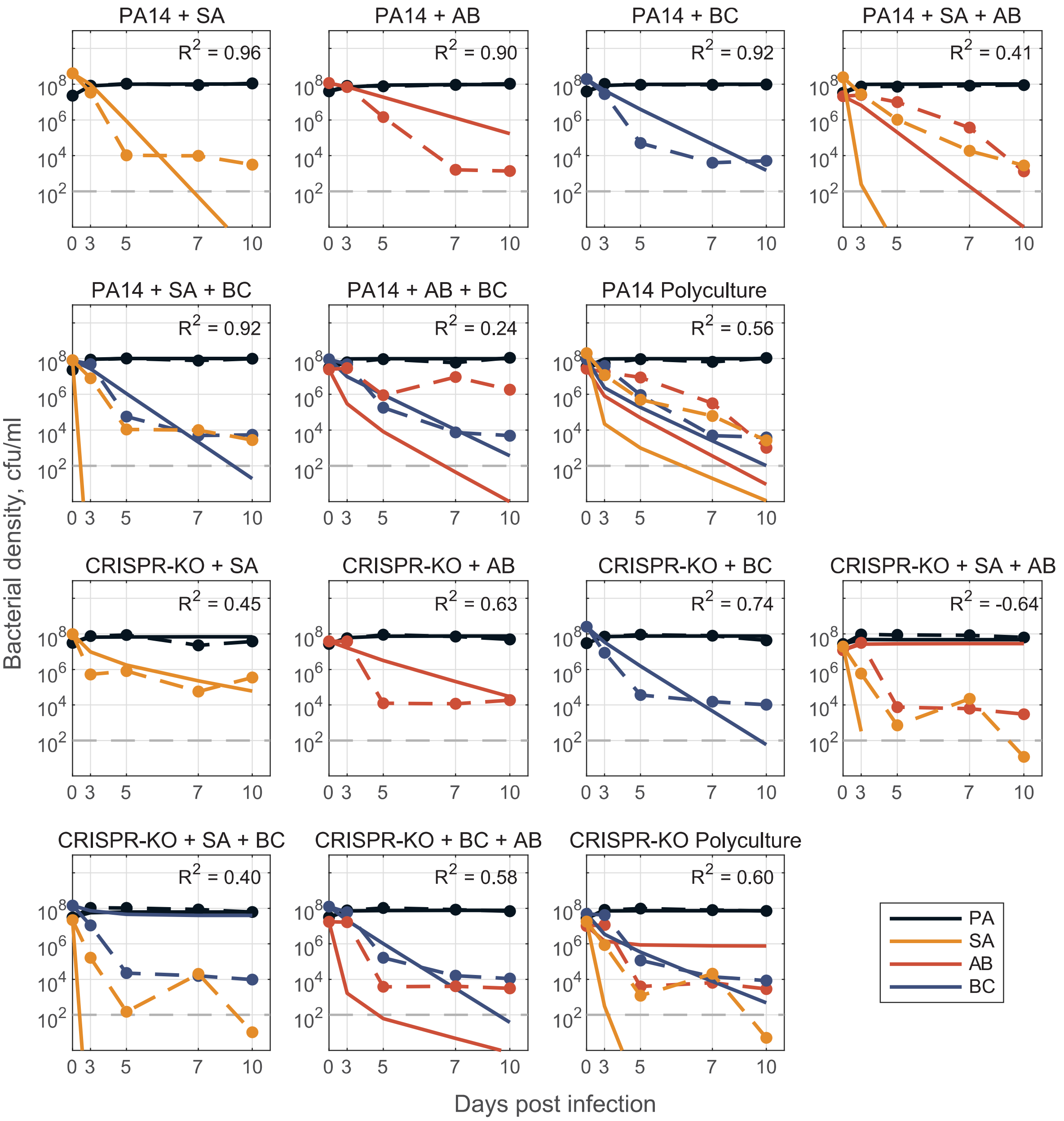

Supplemental Fig 8. Model from no phage data, trained on only pairwise experimental data. Model fit predictions for two-, three-, and full four species community dynamics (solid lines) compared to experimental data (dashed lines). Models were parameterized via optimization with least-squares to fit a system of ODEs (defined as a generalized Lotka-Volterra competition model with n species, where n=1,2,3,4). We parameterize the models via fitting of 1- (for growth rates r_i) and 2- (for all possible

bioRxiv preprint doi: https://doi.org/10.1101/2023.09.26.559468; this version posted September 26, 2023. The copyright holder for this preprint (which was not certified by peer review) is the author/funder, who has granted bioRxiv a license to display the preprint in perpetuity. It is made available under aCC-BY 4.0 International license.

pairwise interaction coefficients $\beta_{i,j}\ \forall i,j = 1,2$) species dynamics and use the resulting coefficients to predict the 3- and 4-species community dynamics. For fitting co-culture data, growth rates r_i were fixed from mono-culture data and interaction parameters $\beta_{i,j}$ were all open. See Methods and Text S1 for a detailed description of mathematical modelling.

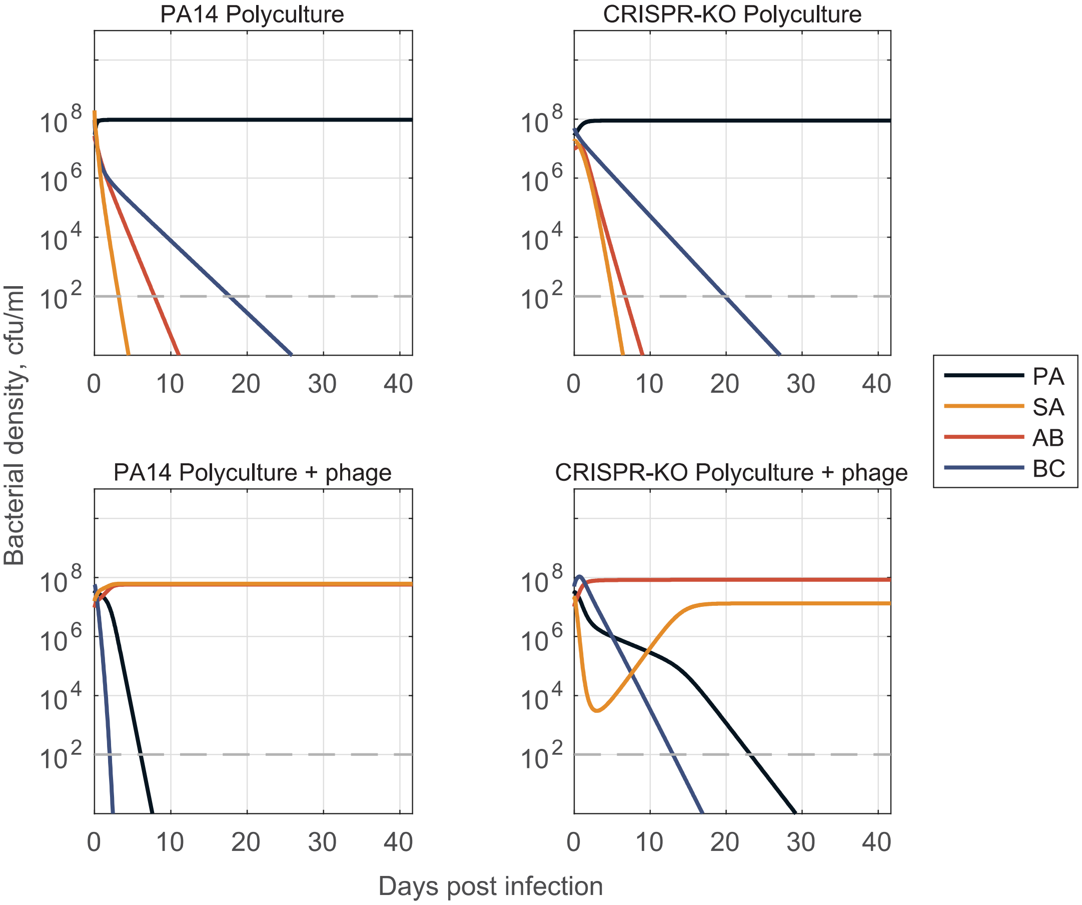

Supplemental Fig 9. Long time simulation of full community model shows shift in ecological outcomes given inclusion of phage. Simulation of the 4-species community gLV model over a long time scale reveals a qualitative shift in the outcome of the community when phage is present. In the absence of phage (top), *P. aeruginosa* is the dominant competitor and only surviving species. In the presence of phage (bottom), the

bioRxiv preprint doi: https://doi.org/10.1101/2023.09.26.559468; this version posted September 26, 2023. The copyright holder for this preprint (which was not certified by peer review) is the author/funder, who has granted bioRxiv a license to display the preprint in perpetuity. It is made available under aCC-BY 4.0 International license.

dominant competitor is eliminated, and we see competitive release of *A. baumannii* and *S. aureus* – maintaining 2 of the 3 non-targeted species in the community. Growth and interaction coefficients for simulation are from the model fits in Figures 7 and 8. For a detailed description of model parameterization and simulation methods, see Methods and Text S1.
